# Relationship of serum lipid profiles in preeclampsia and normal pregnancy, Bangladesh

**DOI:** 10.4314/ahs.v22i2.55

**Published:** 2022-06

**Authors:** Umme Salma

**Affiliations:** Department of Obstetrics and Gynaecology, College of Medicine, Jouf University, Sakaka, Kingdom of Saudi Arabia

**Keywords:** Preeclampsia, normal pregnancy, lipid profile, basal body mass index

## Abstract

**Objective:**

The purpose of this present study to investigate the lipid profile levels and basal body mass index in preeclampsia and normal pregnancy in Bangladeshi women.

**Material and methods:**

This case-control study was conducted at Sheba Hospital Kaligonj Bangladesh with 70 participants among 35 normal pregnancy (control) and 35 preeclampsia women (case) were enrolled from August 2018 to July 2019. Blood samples were obtained for analysis of total cholesterol, triglyceride and high-density lipoprotein by enzymatic assays and low-density lipoprotein by using Fried Ewald's formula in between 22–36 weeks of gestation.

**Results:**

This study found the mean age of preeclampsia and normal pregnancy women were 24.71±2.56 and 23.09±2.1 respectively with significant (P= 0.005). Basal body mass index, total cholesterol, triglyceride and low-density lipoprotein significantly higher (P=0.002), (P= 0.000), (P= 0.022) and (P=0.000) in preeclampsia compared to normal pregnancy respectively. While high-density lipoprotein comparatively lower in preeclampsia than normal pregnancy and consider significant as (P=0.037).

**Conclusion:**

Abnormal lipid profile and increased body mass index is contributed to the development of preeclampsia. The frequent antenatal monitoring of lipid profiles provides the status which helps to require management and reduces the preeclampsia which enhances maternal and fetal wellbeing and fetal outcome.

## Introduction

Preeclampsia means first-time onset of hypertension (blood pressure 140/90 mmHg or more) and protein urine (≥300 mg/day) after 20 weeks of gestation^1^,^2^. Approximately 5–10% causes of fetal and maternal motility and morbidity^3^. The etiology of Preeclampsia due to the absence of trophoblastic invasion in the spiral arteries which leads to uterine arteries more vascular resistance and associated with decline placental perfusion^4^, ^5^. The circulation of preeclampsia women carries the placental products which provoke the endothelial dysfunction, therefore it may development of cardiovascular diseases^6^. Conversely altered lipid profile is strongly associated with preeclampsia women because of endothelial dysfunction. It has been well documented that increased total cholesterol, triglyceride and low-density lipoprotein and decrease high-density lipoprotein has direct link to preeclampisea^7^,^8^. Therefore, the aim of this study to investigate the serum total cholesterol, triglyceride, low-density lipoprotein and high-density lipoprotein for the relationship between preeclampsia and normal pregnancy in Bangladeshi women.

## Methods

This case-control study was carried out at Obstetrics and Gynecology department of Sheba Hospital Kaliganj, Bangladesh, over a period from August 2018 to July 2019. A total of 70 pregnant women were agreed to participate after explained the purpose and objective of this study. The present study collected written consent of each participant with ethically approved from Hospital clinical research committee. All pregnant women were divided into two groups such as normal pregnancy women for the control group (n=35) and preeclampsia women for case group (n=35), who were visited for antenatal follow-up in outpatients or require admitting inpatients. The diagnosis of preeclampsia by the first-time onset of systolic blood pressure ≥ 140 mmHg and diastolic blood pressure ≥90 mmHg after 20 weeks of gestation and associated with proteinuria, if urinary protein excretion ≥300 mg/24 hours. The inclusion criteria of the present study were included that all pregnant women were primigravida in both groups, age range from 19–29 years, having 22–36 gestational weeks with a single live fetus. While multiple pregnancy, essential hypertension, diabetes mellitus and renal disease were excluded. We noted detailed obstetrical history and examination findings of both control and case groups. The recorded body mass index (BMI) of both groups of all pregnant women followed by WHO calculations such as normal, overweight, and obesity range were considered 18.5 to 24.9 kg/m^2^, 25–29.9 kg/m^2^, and> 30 kg/m^2^ respectively. The Blood pressure measure by mercury sphygmomanometer and repeated the procedure after 5 and 10 min of each pregnant woman which was recommended by the American Heart Association^9^. Total 5ml to 8ml blood drawn from the antecubital vein with aseptic precautions then put in ethylene diamine tetra acetic acid-containing vials for analysis of lipid profiles like total cholesterol (TC), triglyceride (TG), high-density lipoprotein (HDL), low-density lipoprotein (LDL), and also complete blood count. The clotted blood sample was centrifuged at 3,000rpm for 10 min and serum keep stored at -80°C until further assayed. The analyzed the serum concentration of total cholesterol, high-density lipoprotein cholesterol and triglycerides levels by the method of an automated system (Biotecnica Chemistry Analyzer 3000, Italy) with standard enzymatic assays, while LDL was measured by using Fried Ewald's formula. All values of lipid profiles were recorded.

## Statistical analysis

Statistically analyzed of all data by using SPSS software 20.00 versions with descriptively, implement the number of pregnant women, mean and standard deviations. As well as apply the Chi-square t-test for data which were a comparison between normal pregnancy and preeclampsia women. Significant was considered at p-value <0.05.

## Results

In the present study, all seventeen pregnant women divided into two groups, the normal pregnancy women (n=35) and preeclampsia women (n=35) with an aged range from 19 to 29 years. The means age 23.09±2.1 and 24.71±2.56 of normal pregnancy and preeclampsia women were respectively and higher statistically significant (P=0.005) in preeclampsia women. (Table 1) represents the clinical characteristic of participants which included basal body mass index (BMI) 23.31±2.8 in normal pregnancy and 25.45±2.82 in preeclampsia women.

**Table 1:** Clinical characteristic of all participants

Lipid profile	Normal pregnancy (n=35)	Preeclampsia (n=35)	P value
TC	170.51±12.56	222.51±11.98	0.000
TG	144.86±5.0	204.54±3.39	0.022
HDL	35.57±3.0	33.97±3.28	0.037
LDL	89.25±14.3	143.03±9.61	0.000

The basal body mass index significantly higher in pre-eclampsia women than normal pregnancy as consider p values 0.002. Mean systolic 139.74±3.78 and diastolic 92.22±2.96 blood pressure was significantly increased in preeclampsia women compared to normal pregnancy (P=0.000). The lipid profiles in preeclampsia women such as total cholesterol (TC) 222.51±11.98 and normal pregnancy were 170.51±12.56 (P=0.000). Triglyceride levels significantly higher in preeclampsia women than normal pregnancy women(P=0.022). Compared to the high-density lipoprotein (HDL) between preeclampsia and normal pregnancy women was 33.97±3.28 and 35.57±3.0 respectively which has lower preeclampsia women and statistically significant (0.037). As well as the present study found low-density lipoprotein (LDL) significantly higher 143.03±9.61 in preeclampsia women than 89.25±14.3 in normal pregnancy women (P=0.000).

The concentrations of all lipid profiles levels of both preeclampsia and normal pregnancy women represent in (Table 2).

**Table 2:** Status of lipid profile in preeclampsia and normal pregnancy

Lipid profile	Normal pregnancy (n=35)	Preeclampsia (n=35)	P value
TC	170.51±12.56	222.51±11.98	0.000
TG	144.86±5.0	204.54±3.39	0.022
HDL	35.57±3.0	33.97±3.28	0.037
LDL	89.25±14.3	143.03±9.61	0.000

Abbreviation:

TC=Total cholesterol, TG= Triglyceride, HDL= High density lipoprotein, LDL= Low density lipoprotein.

## Discussion

In this present study, we investigated the lipid profile levels of preeclampsia and normal pregnancy in Bangladeshi women. The present study compared 35 preeclampsia women with 35 normal pregnancy women along age range 19–29 years. The relation of age in thepreeclampsia women found was highly significant than normal pregnancy women. Previously reported that altered lipid profile has the link to increased the age in preeclampsia women^10^,^11^. The basal body mass index (BMI) in preeclampsia women in this study was shown higher significant compared to normal pregnancy women and consider at (P= 0.002), which was similarly reported to another study.10 Perhaps the elevated of BMI in preeclampsia women may probable causes increased insulin resistance and as well as the circumstance of inflammation associated with overweight and obesity^12^. However, the excessive body weight of pregnant women individual itself responsible for the development of endothelial dysfunction of the placenta which may lead cause of preeclampsia. Conversely, the endothelial dysfunction consider as an important fact for the mechanism of increased blood pressure during pregnancy and altered lipid profiles also make a key role for the endothelial dysfunction^13^,^14^. The Present study also found high levels of serum triglycerides (TG) and total cholesterol (TC) in preeclampsia women compared to normal pregnanyc respectively and statistically significant (P= 0.022) and (P= 0.000) which were found other studies also^15^. High levels of cholesterol may encourage the production of free radicals which has direct relation of atherosclerosis and associated abnormal lipid profile consider as uncertainly to the development of preeclampsia. Nevertheless, increased triglyceride (TG) in preeclampsia has indicates that the relation of insulin resistance because of the excessive body weight of women. While it has been consider that increased triglyceride is necessary for the development of peeclampsia or risk for pre-eclampsia.

There was evidence in preeclampsia women that elevated serum concentration of triglycerides has a relationship with systolic and diastolic blood pressure in pressure^16^. It has been reported that increased maternal triglyceride levels perhaps significant of pre -existing hyperlipidemia and considered as pathogenesis of preeclampsia which was supported to our results 17. A recent study also suggested, that there was a positive correlation between high maternal serum lipid and preeclampsia 18.

Additionally, the present study found increased low-density lipoprotein(LDL) in preeclampsia women contrast to normal pregnancy and consider as significant (P=0.000). While the previous study has evidence that elevated LDL act as a risk factor for atherosclerosis^19^. While elevated estrogen and progesterone in preeclampsia may be supposed causes to raise LDL levels in preeclampsia women. Additionally, there was demonstrate of LDL may perhaps elevate the arterial sensitivity to a pressor agent that suppresses the endothelium dependant vasodilatation and development of dysfunctional endothelium. Therefore, it has produced pathology in glomerular of the kidney which consequently leakage of protein in the urine that reveal the severity of preeclampsia 20. Besides, the present study found decreased high-density lipoprotein (HDL) in preeclampsia women compared to normal pregnancy women (P=0.037). While decreased HDL and increased TG has contributed to the development of cardiovascular complication during pregnancy. On the other hand, insulin resistance may cause to reduce the HDL in preeclampsia women. However, in the present study found increased LDL and decreased HDL in preeclampsia women may consider as a risk factor for the chance of atherosclerosis^21^.

## Conclusion

The finding results of the present study indicate that abnormal lipid profile such as increased serum concentration of TC, TG, LDL and decreased HDL may contribute for the development of preeclampsia. Besides, basal body mass as an individual key factor for preeclampsia. Consequently, routinely antenatal monitoring of lipid profile may reduce the possibility of preeclampsia which enhances maternal and fetal well-being and fetaloutcome. However, detection and adjust of lipid profile during pregnancy can help for better management and prevention of preeclampsia.
